# Supplementary material for: Are carbon emissions trading and green financial instruments synergistic? -Comprehensive quantitative research based on content analysis
Source: PLoS One. 2024 Mar 7;19(3):e0298601. doi: 10.1371/journal.pone.0298601 (PMC10919601; doi:10.1371/journal.pone.0298601)
Supplement: S1 Data — (ZIP) [file pone.0298601.s001.zip › Data/地级市Z_F工作报告.zip!/┴╔─■/╠·┴δ╩╨/└┤╫╘╣1⁄2╓┌║┼í╛┬φ┐╦╩2╛▌═°í┐_2011.docx]

各位代表：

　　现在，我代表市人民政府向大会报告工作，请予审议，并请市政协委员和其他列席会议的同志提出意见。

　　一、2010年暨“十一五”工作回顾

　　过去的一年，我们在省委、省政府和市委的正确领导下，团结和带领全市各族人民，锐意进取，奋力拼搏，全面完成了市六届人大二次会议确定的各项任务目标。

　　——对外开放取得重大成果。引进域外投资项目990个，到位资金663亿元。实际利用外资2.6亿美元，增长52.9%。外贸出口5亿美元，增长20%。奥特莱斯现代服务产业区、帽山风景旅游度假区等一批项目正式启动；曙光专用车、檀香湾五星级酒店等一批项目开工建设；冀东专用车、中利科技等一批项目建成投产；铁岭电厂二期、清河电厂上大压小二期工程获国家发改委核准；古榆煤田勘探取得明显进展。

　　——工业经济实现新跨越。沈铁工业走廊支撑作用日益显现，占全市工业经济总量的比重达到75%。工业结构明显优化，先进制造业成为第一大主导产业。产业集群发展壮大，专用车生产基地、换热设备产业基地进入全省十大新型工业化示范基地。工业企业做大做强，销售收入亿元以上企业发展到260户，其中10亿元以上企业36户。工业企业实现主营业务收入2200亿元，增长56%。实现高新技术产品产值240亿元，增长50%。

　　——城镇化进程快速推进。铁岭新城建成区面积达到10平方公里，入住人口10万人。医院、学校、市场等重点公用服务工程交付使用，恒基地产、新弘国际城等一批商住工程开工建设，桂林公园、莲花湖西扩等生态建设工程相继竣工。铁岭新城成为唯一入选上海世博会主题馆的中国城市，受到全世界的广泛关注和高度赞誉。在联合国人居署、国家住建部、上海市政府联合举办的世界人居日大会上，又荣获中国人居环境范例奖。改造银州区的十件实事全部完成，管理体制进一步理顺。数字城市建设成效明显，铁岭一卡通荣获全国智能卡行业最高奖。沈铁二号公路铁岭段竣工通车，新台子、腰堡新市镇开工建设，沈铁共享024区号获得国家工信部批复。开原滨水新城、昌图湖滨新城建设全面启动。全市城镇化率达到47%。

　　——县域经济迈上新台阶。开原市一举进入全国百强县，调兵山市、铁岭县跻身全省十强，昌图县、西丰县在全省位次前移。四个100万亩工程如期实现，畜牧业产值占农林牧渔业总产值的比重提升到56.7%，农业产业化率达到53%，我市被授予“中国榛子之都”。农业基础设施建设得到加强，有望连续6年夺得省大禹杯。完成植树造林43万亩，森林覆盖率达到34.3%。农机总动力211万千瓦，农业机械化水平达到56.6%。粮食总产量在大灾之年仍然达到71亿斤，成为历史上第二个丰收年。

　　——现代服务业呈现勃勃生机。东北物流城一期项目投入运营，二期工程全面开工建设，完成投资30亿元。北方金融后台服务基地建设取得新进展。职业教育基地初具规模，在校生达到5万人，技师学院、铁岭卫校成功晋升高等职业学院。会展经济开始起步，成功举办了专用车博览会等5次全国性大型展会。城区经济健康发展，提前完成三年倍增计划。月星国际、居然之家等一批品牌企业开业，粤海五星级酒店交付使用。房地产业健康发展，新开发面积1066万平方米。旅游业方兴未艾，成功举办了生态休闲旅游节。万村千乡市场工程、农村新网工程和新型科技储粮仓工程扎实推进。

　　——财税金融工作实现新突破。地方财政一般预算收入增幅继续保持全省领先，总量在全省位次继续前移。组织国税收入35.1亿元、地税收入51亿元，分别增长29.4%和48%。向上争取各类无偿资金70亿元，增长11.8%。金融机构各项存款余额632亿元，贷款余额484亿元，分别增长10.5%和14.1%。市商业银行增资扩股获国家银监会批准，调兵山村镇银行挂牌营业，西丰村镇银行获省银监局筹建批复，小额贷款公司发展到19家。企业上市实现历史性突破，东北物流城、新兴佳波纹管分别在纽约证交所和纳斯达克成功上市。获得土地出让金19.5亿元、国有资产整合收入5.7亿元，走出了一条资源资产化、资产资本化、资本证券化的融资新路子。

　　——节能减排成效显著。关停取缔20家严重污染环境企业，对71家企业进行了限期治理，7座污水处理厂投入运行。万元生产总值综合能耗、水耗分别下降3%和2%，二氧化硫和化学需氧量排放量分别减少8%和1%。城市绿化覆盖率达到39%，铁岭新城空气质量达到森林和海洋水平。辽河生态治理走在全省前列，一级支流率先消灭超五类水体。

　　——民生工程逐项落实。筹集资金2.2亿元，开展实名制就业培训39639人，安置实名制就业71512人，零就业家庭实现动态为零。筹集资金19.2亿元，确保了企业养老金按时足额发放并提高了保障标准。筹集资金2.5亿元，确保了167547名城乡低保对象的基本生活。筹集资金2894万元，为119807名城乡困难群众发放了医疗救助金。筹集资金6072万元，妥善安置了8.35万名受灾群众。筹集资金2943万元，使13623名农村五保对象实现了应保尽保。筹集资金7182万元，保障了11600名优抚对象的基本生活。筹集资金5.3亿元，解决了10208户低收入家庭住房困难问题。筹集资金9414万元，实施了银州区铁西一铁道门和电子厂平房区改造工程。筹集资金1837万元，改造老旧住宅小区72万平方米。筹集资金3200万元，改造农村低保边缘户D级危房2000户。筹集资金8497万元，解决了16.4万名农村人口饮水安全问题。

　　——社会事业全面进步。科技创新体系逐步完善，全民科技素质进一步提升。铁岭高中新校区、铁岭新城实验中小学交付使用，铁岭县莲花中学如期开学，开创了城乡一体、均衡发展的办学新模式。普九工作高分通过省验收，千人支教计划顺利实施，校园安全得到保障。新农合运行机制更加完善，社区卫生服务覆盖面继续扩大，各类重大传染病得到有效防控，爱国卫生运动深入开展。秧歌戏《杨柳情》在省第八届艺术节上喜获金奖，文化信息资源共享工程和有线广播电视数字化同步推进，文物保护工作卓有成效，群众文化活动亮点纷呈。体育运动蓬勃开展，连续5年被国家体育总局评为全民健身活动优秀组织奖。人口和计划生育工作得到加强，保持了低生育水平。

　　——社会保持和谐稳定。公安执法规范化、治安防控信息化建设全面加强，五五普法工作取得显著成效，社会治安保持稳定。安全生产形势总体平稳，信访总量稳步下降，食品药品安全得到保障。应急管理工作体系全面建立，处置突发事件能力明显增强。民族关系和谐融洽，宗教活动依法有序。政府法制工作得到加强，依法行政水平进一步提高。公共行政服务四级联动新机制全面启动，在全省率先实现了行政审批和行政事业性收费目录管理。政务公开工作不断深化。国防动员和后备力量建设得到加强，进一步巩固了全国双拥模范城创建成果。群众性精神文明创建活动广泛开展，社会文明程度明显提升。自觉接受市人大的依法监督和市政协的民主监督，人大议案、代表建议、政协提案办理工作质量不断提高。

　　外事、侨务、台办、统计、工商、审计、物价、综合执法、档案、气象、电业、规划、出入境检验检疫、烟草、人防、国家安全、武警、消防、新闻出版、地震、政务接待、残联、科协、商业保险、邮政、通信、驻外联络、市志、老龄、仲裁等工作也都取得了新成绩。

　　随着2010年各项任务的完成，“十一五”也画上了一个圆满句号，铁岭进入了赶超发展新阶段。预计2010年，全市生产总值实现720亿元，是“十五”末期的2.7倍。财政一般预算收入实现80.1亿元，是“十五”末期的6.3倍。规模以上工业增加值实现400亿元，是“十五”末期的4.8倍。全社会固定资产投资完成1000亿元，是“十五”末期的9.3倍。社会消费品零售总额实现227亿元，是“十五”末期的2.4倍。城镇居民人均可支配收入1.4万元，是“十五”末期的2.2倍。农村居民人均纯收入7570元，是“十五”末期的2倍。财政一般预算收入、规模以上工业增加值在全省的位次提升到第六位，全社会固定资产投资提升到第五位。三次产业结构比由“十五”末期的26.4：40.5：33.1调整到18：57：25。工业增加值占生产总值的比重由“十五”末期的36.2%提高到55.6%。财政一般预算收入占生产总值的比重由“十五”末期的4.8%提高到11%。税收占财政一般预算收入的比重由“十五”末期的68.2%提高到72.4%。

　　过去的五年，铁岭大地发生了沧海桑田般的变化，取得了令人瞩目的巨大成就。以经济总量在全省位次前移为标志，实现了超常规跨越式发展，赶上了时代发展的新潮流。以沈铁工业走廊迅速崛起为标志，结束了传统农业地区的历史，开辟了工业化的新纪元。以铁岭新城拔地而起为标志，实现了由中等城市向现代化大城市的跨越，走进了城镇化的新时代。以开原市进入全国百强县为标志，谱写了工业化、城镇化和农业现代化协调发展的辉煌篇章，实现了县域经济的新突破。以东北物流城和奥特莱斯项目落户为标志，建设了一大批特色鲜明的重点工程，形成了现代服务业竞相发展的新局面。以成功引进益海嘉里、中航工业集团、恒基兆业等世界500强企业为标志，构建了全方位、多层次、宽领域的对外开放格局，踏上了与巨人同行的新征程。

　　过去的五年，306万铁岭人民波澜壮阔的实践可歌可泣，励精图治的经验弥足珍贵，高瞻远瞩的谋划影响深远。由于我们紧密结合铁岭实际贯彻落实科学发展观，以工业化带动城镇化，以城镇化促进工业化，努力破解“一小两低”难题，走出了一条欠发达地区赶超发展的成功之路。由于我们紧紧抓住重要战略机遇期，高举对外开放大旗，连续开展项目年活动，引进建设了一批重大项目，为铁岭经济社会发展注入了强大动力。由于我们牢牢把握发展是硬道理，持续推动经济增长，同步提高质量效益，不断积蓄发展后劲，为未来一个时期经济社会发展再上新台阶奠定了坚实基础。由于我们善于破解制约发展的瓶颈问题，大胆创新体制机制，在深化改革中激发活力，在拼搏进取中拓展空间，为经济社会又好又快发展积累了丰富经验。由于我们时刻牢记以人为本、执政为民，在发展经济的同时，更加注重发展社会事业和改善民生，在发展社会事业中讲政绩，在实施民生工程中树形象，为改革发展营造了稳定和谐的社会环境。由于我们始终坚持解放思想、转变观念不动摇，广大干部群众的精神面貌发生了深刻变化，大胆探索、宽容失误，崇尚改革、鼓励创新，全市上下政通人和谋发展、万众一心促和谐的局面已经形成，为全面建设小康社会提供了取之不尽、用之不竭的力量源泉。在此，我代表市人民政府，向辛勤工作在各条战线上的全市各族人民，向各位人大代表、政协委员，向各民主党派、工商联、无党派人士及人民团体，向中省直驻铁单位，向驻铁部队指战员、武警官兵，向所有关心、支持、参与铁岭改革开放和现代化建设的海内外朋友表示衷心的感谢并致以崇高的敬意！

　　在看到成绩的同时，我们也清醒地看到目前存在的主要矛盾和问题。一是经济发展方式还比较粗放，自主创新能力不强，新兴产业发展还不充分，节能减排和环境保护压力较大。二是发展不平衡问题比较突出，城乡之间、县域之间在发展速度、发展质量、基础设施等方面还存在较大差异。三是居民收入水平还比较低，没有完全做到与经济发展同步，全市城乡还有相当数量的困难群众。四是政府职能转变还不适应新形势、新任务的要求，政府自身建设还有不尽人意之处。这些问题已经引起我们的高度重视，并将在今后的工作中采取有力措施认真加以解决。

　　二、“十二五”指导思想和发展目标

　　“十二五”时期是全面建设小康社会的关键时期，是深化改革开放、加快转变经济发展方式的攻坚时期。全市经济社会发展的指导思想是：以邓小平理论和“三个代表”重要思想为指导，深入贯彻落实科学发展观，以科学发展、创新发展、和谐发展为主题，以加快经济发展方式转变和社会管理模式转型为主线，以改革开放和科技创新为动力，以保障和改善民生为出发点和落脚点，深入实施开放立市、工业强市、三产活市、科教兴市、城乡统筹、全民创业六大战略，全力推进工业化、城镇化双轮驱动，全面实现“四个跨越”，推进新铁岭大铁岭建设跨入新阶段，努力把我市建成实力铁岭、活力铁岭、魅力铁岭、幸福铁岭。

　　主要发展目标是：

　　——综合经济实力进入全省前5名。生产总值年均增长18%，财政一般预算收入、规模以上工业增加值、全社会固定资产投资年均增长20%以上。三次产业结构比调整到10：55：35。

　　——建成100万人口的中心城市。铁岭新城35平方公里全部建成，人口达到35万人；银州区城市功能全面提升；新台子、腰堡、凡河三个新市镇基本建成，集聚人口分别达到10万人；中心城区面积达到100平方公里，生产总值实现1000亿元，财政一般预算收入实现100亿元，建成独具魅力的生态之城、宜居之地、时尚之都，成为名副其实的中国北方水城。开原滨水新城、昌图湖滨新城基本建成，调兵山市实现城乡一体化，西丰县城面貌明显改观。全市城镇化率提高到60%以上。

　　——新型工业化基地基本建成。专用车生产基地实现销售收入1000亿元以上，把铁岭打造成中国汽车城。换热设备、起重机械、新能源装备和有色金属产业集群销售收入分别实现500亿元以上，其它产业集群销售收入实现100亿元以上。开发区、高新区进入国家级开发区行列。先进制造业占工业总量的比重提高到50%以上。

　　——县域经济再上新台阶。调整优化农业结构，基本形成一县一品新格局，把我市建成辽宁省畜牧产业第一市。开原市在全国百强县位次明显前移，铁岭县、调兵山市进入全国百强县，昌图县进入全省中上游行列，西丰县摆脱贫困面貌。

　　——现代服务业基地基本建成。东北物流城、奥特莱斯现代服务产业区、帽山风景旅游度假区、北方金融后台服务基地和职业教育基地全部建成，把铁岭打造成依托沈阳、连结哈大、辐射东北的现代服务业基地。现代服务业占服务业比重提高到50%以上。

　　——人民生活水平显著提升。城镇居民人均可支配收入达到全省平均水平，农村居民人均纯收入超过全省平均水平。建立健全覆盖城乡的社会保障体系和基本公共服务体系。居民幸福指数明显提高。

　　做好“十二五”时期的工作，要牢牢把握以下四个着力点：

　　一是始终坚持发展第一要务。坚持以增量带动结构优化，继续保持较高的发展速度，着力做大经济规模，提升经济总量，为经济结构战略性调整拓展空间、创造条件。

　　二是始终坚持固定资产投资和工业经济两大重点。继续保持固定资产投资和工业经济快速增长的良好势头，拉动经济发展，增加财政收入，促进城乡就业。

　　三是始终坚持把构建现代产业体系作为主攻方向。做大做强先进制造业，使其成为我市的支柱产业，这是构建现代产业体系的首要任务。大力发展战略性新兴产业，培育新的经济增长点，这是构建现代产业体系的重要依托。加快发展现代服务业，推进服务业与制造业深度融合，这是构建现代产业体系的发展方向。全面发展现代农业，促进农业增产、农民增收，这是构建现代产业体系的重要基础。

　　四是始终坚持把工业结构调整作为重中之重。用高新技术和先进适用技术改造提升传统产业，引导企业把技术改造与产业升级结合起来，推动工业经济总体水平跃上一个新台阶。优化中小企业发展环境，支持企业加快结构调整，向专、精、特、新和产业集聚方向发展。

　　三、2011年的主要工作

　　今年是实施“十二五”规划的开局之年，做好今年的各项工作具有十分重要的意义。

　　主要预期目标是：生产总值实现860亿元，增长18%。财政一般预算收入实现96亿元，全社会固定资产投资完成1200亿元，规模以上工业增加值实现480亿元，分别增长20%。社会消费品零售总额实现267亿元，增长17.5%。城镇居民人均可支配收入实现1.6万元，农村居民人均纯收入实现8700元，分别增长15%。城镇登记失业率控制在3.8%以下。

　　为实现上述目标，重点抓好以下10项工作：

　　（一）高举对外开放大旗，深入开展项目年活动

　　继续加大招商引资力度。以东北、长三角、珠三角、香港等地区为重点，以沈铁工业走廊为载体，强化产业集群招商、主题概念招商，组织好境内外系列招商活动，确保引进亿元以上项目120个，引进内资780亿元，实际利用外资5亿美元，600个项目开工建设，300个项目投产达效。大力拓展东欧、中东、南美等新兴市场，确保外贸出口实现6亿美元。依托专用车博览会、换热设备博览会、榛子节等常态化载体，大力发展会展经济。

　　（二）以实施“五项工程”为重点，推动工业经济优化升级

　　发展壮大产业集群。专用车生产基地确保50个项目建成投产，实现主营业务收入300亿元。换热设备产业基地确保80个项目建成投产，起重机械生产基地新开工项目30个。重点抓好冀东专用车、博大生物等400个技改项目，做大做强骨干企业，销售收入亿元以上企业发展到320户，10亿元以上企业发展到50户。实现高新技术产品产值340亿元。全力推进益海嘉里生物化工、铁煤集团60万千瓦煤矸石发电等战略性新兴产业项目建设。进一步优化发展沈铁工业走廊，完善园区功能，建立健全园区服务体系。继续加大节能减排力度，万元生产总值综合能耗和水耗分别下降5%，二氧化硫和化学需氧量排放量分别减少2%。

　　（三）以中心城市建设为重点，加快推进城镇化进程

　　加快铁岭新城建设，进一步完善城市功能。集中力量抓好凡河南岸基础设施、集中供热管网建设，推进恒基兆业天水河商业步行街和莲花湖高档住宅等重点工程建设，完成投资100亿元。全面推进新台子、腰堡、凡河新市镇开发建设，开工建设沈铁城际铁路、沈四高速公路铁岭段拓宽改造等工程。继续改造提升银州区功能，重点办好十件实事。1.扩建柴河垃圾处理厂，开工建设渗漏液处理工程。2.建设光荣街带状游园。3.完善龙山风景区服务功能。4.逐步拆除10吨以下小锅炉，实行集中供热。5.整治燃气管道安全隐患。6.实施污水处理厂淤泥处理工程。7.建立水源地保护区，清理自备井，改造、整合二次加压供水设施。8.改造城市污水管网。9.改造小街小巷基础设施，对老旧住宅小区环境进行综合整治。10.解决廉租住房2382户。进一步理顺中心城区管理体制，加快数字城市和生态城市建设，全力争创国家环保模范城和国家卫生城。加快推进开原滨水新城、昌图湖滨新城建设。进一步放宽外来人口落户条件，加快城镇人口集聚步伐。

　　（四）抓好农业农村工作，做大做强县域经济

　　加快保护地蔬菜、畜牧、榛子三大主导产业开发，新增保护地蔬菜10万亩、榛子6万亩，畜牧业产值达到228亿元。继续抓好铁南都市农业、开西现代农业、昌北现代畜牧业、西丰特色产业四大示范区建设，发展壮大开原苗木花卉、昌图花生等特色产业基地，加快一县一品步伐。大力发展农业产业化经营，农业产业化产值增长20%。搞好以水利设施为重点的农业基础设施建设。促进土地流转和耕作方式转变，农业机械化水平提高到59%。深入开展“绿化杯”竞赛活动，完成人工造林29万亩，全面恢复辽河生态。加快农民专业合作社建设，大力开展有组织的劳务输出，解放和转移农村劳动力。创新农业科技服务模式，深入开展科技特派活动。以完善基本公共服务体系为重点，扎实推进社会主义新农村建设。进一步壮大县域经济实力，开原市在全国百强县实现位次明显前移，铁岭县、调兵山市全力向全国百强县冲刺，昌图县、西丰县在全省位次继续前移。

　　（五）全力抓好重点项目，加快构建现代服务业体系

　　全面推进铁岭新城现代服务业基地和沈铁工业走廊服务产业带建设。抓好东北物流城二期工程建设，实现销售收入30亿元。举全市之力支持奥特莱斯项目建设，确保2012年投入运营。加快北方金融后台服务基地建设，增强区域金融资源吸引力。扩大职业教育基地规模。大力发展旅游产业，实现旅游业总收入100亿元。促进房地产业快速健康发展，新开发房地产面积1200万平方米。启动银州区寿光果蔬贸易城、开原大商新玛特、西丰鹿茸中草药材期货市场和昌图滨河商业金廊建设，东北机动车交易市场、调兵山檀香湾五星级酒店投入使用。扎实推进万村千乡市场工程、农村新网工程和新型科技储粮仓工程，改善农村消费环境，促进农村消费结构升级。

　　（六）采取有力措施，破解发展瓶颈

　　搞好新一轮土地利用总体规划修编，深入开展城乡建设用地增减挂钩工作，充分挖掘存量建设用地潜力，满足发展建设需要。强化供地用地监管，对多批少用、批而不用等闲置低效用地进行劝退，对土地违法违规案件实施严格的问责制度。推动招商引资向招商选资转变，提高投资强度和容积率，实现集约节约用地。继续坚持资源资产化、资产资本化、资本证券化融资工作思路，充分挖掘融资潜力，探索新的融资途径，形成多元化、广渠道融资新格局。积极与国家政策性银行、国有商业银行开展对接，发挥其融资主渠道作用。做大做强商业银行、村镇银行等地方金融机构，积极引进股份制银行，使其成为融资的重要补充。规范县级融资平台管理，壮大融资平台实力，整合县域公共资源，优化平台财务状况和资产结构，为对接资本市场奠定基础。通过股权融资、发行企业债券、中期票据、利用产业基金和私募基金、企业上市等途径，开辟新的融资渠道，努力破解资金瓶颈。加快推进社会信用体系建设，努力打造诚信铁岭。

　　（七）着力保障和改善民生，维护社会公平正义

　　筹建人力资源和社会保障服务中心，重点做好高校毕业生、农村转移劳动力、城镇就业困难人员和退役军人就业工作，安置实名制就业3.8万人。做好养老保险扩面征缴工作，确保养老金按时足额发放。继续扩大医疗保险覆盖范围，重点做好困难企业职工、在校学生和城镇居民的参保工作。提高城乡低保标准，落实城乡医疗救助、城区低保对象取暖补贴等各项政策。妥善安置受灾群众，对易遭受洪涝灾害的村屯实施整体迁移。改造农村低保边缘户D级危房2000户。开工建设妇女儿童活动中心、特困家庭危重病人托管中心、儿童福利院、流浪未成年人救助保护中心。新建农村饮水安全工程70处，解决7万人饮水安全问题。切实抓好“菜篮子”、“菜园子”工程，保障市场供给。全面加强价格调控监管工作，保持物价总体水平基本稳定。

　　（八）繁荣发展各项社会事业，建立健全基本公共服务体系

　　搭建创新体系平台，扶持科技型企业发展，完善产学研合作机制，提升自主创新能力。深入开展教育年活动，重点实施十二大工程。完成铁岭师专晋升本科任务。改扩建农村标准化中心幼儿园20所，建设农村九年一贯制学校10所。推广铁岭县莲花中学办学模式，扩大农村学生到城市就学比例，促进教育公平。推进职业教育集约特色发展，积极争创国家级重点学校、重点专业和重点实训基地。加大校舍维修改造和校园周边环境整治力度，确保校园安全。抓好重大人才工程，推动人才事业全面发展。促进基本公共卫生服务均等化，启动国家基本药物制度，加快县级医院改扩建步伐，抓好中医院搬迁、妇婴医院扩建工程，进一步完善社区卫生服务体系和新农合制度，切实改善城乡居民医疗条件。抓好重大传染病防治工作，确保不发生大的疫情。推进文化体制改革，发展壮大文化产业，实施乡镇文化广场、农家书屋、广播电视村村通、文化信息资源共享、有线广播电视数字化等文化惠民工程，争取银冈书院等国家级重点文物保护单位申报成功，抓好辽河博物馆、城市地球馆等项目建设。办好市七运会，抓好铁岭新城“一场两馆”建设。开展人口和计划生育综合改革示范县区创建活动，综合治理出生人口性别比偏高问题。加大双拥工作力度，增进军政军民团结。不断拓展城乡共建范围，广泛开展群众性精神文明创建活动，大力提升公民的文明素质。

　　（九）加强和创新社会管理，维护社会和谐稳定

　　构建社会管理新格局，推进管理重心下移，强化基层社会管理职能。加强信访工作，建立健全社会调解工作机制，妥善协调各种利益关系，维护群众合法权益。推进应急平台体系建设，完善应急救援联动机制，妥善处置各类突发事件。实施城乡统一的户籍登记制度，探索建立人口管理服务新模式。推进警务体制机制改革，建成现代警务指挥体系。严厉打击各种违法犯罪活动，建立健全社会治安防控体系，建设横向集成、纵向贯通、互联互通、高度共享的社会管理信息平台，提升虚拟社会管控能力，继续争创全省最稳定地区。全面启动“六五”普法，强力推进依法治市。落实宽严相济的刑事政策，认真开展社区矫正工作。进一步强化安全生产责任体系，实施企业安全生产标准化达标工程，建立企业隐患排查治理常态化制度，杜绝重特大生产事故。健全食品药品安全监管体制，确保食品药品质量安全。全面贯彻党的民族政策，促进少数民族地区经济社会加快发展。依法加强对宗教事务的管理，规范宗教活动。

　　（十）加快转变政府职能，努力建设法治政府和服务型政府

　　加强民主政治建设。自觉接受人大依法监督和政协民主监督，坚持重大事项向人大报告、向政协通报制度，认真执行人大及其常委会的各项决议、决定，积极支持人民政协履行职能，认真办理人大议案、代表建议和政协提案，加强与民主党派、工商联、无党派人士、人民团体和社会各界人士的联系。进一步畅通群众诉求渠道，努力做到民主决策、科学决策、依法决策。全面推进依法行政。深入贯彻依法行政实施纲要，健全依法行政各项制度。加强规范性文件监督管理，提高制度建设质量。深化行政执法体制改革，推进执法重心下移，避免多头执法、交叉执法。健全监督制约机制，规范行政执法行为。加快转变政府职能。继续精简和下放行政审批事项，全面推广公共行政服务四级联动新机制和全天候服务模式，努力打造服务高地、成本洼地、发财宝地。切实加强机关作风建设。强化政务督查和绩效考评，狠抓工作落实，确保件件有着落，事事有回音，提高政府公信力和执行力。克服官僚主义、形式主义，大力提倡开短会、讲短话、发短文，做到查实情、说实话、办实事。加强反腐倡廉建设。严格执行党风廉政建设责任制和领导干部廉洁从政准则，推行行政首长经济责任问责制度，大力推进政务公开和行政权力公开透明运行，建立统一的公共资源交易中心，规范公共资源交易行为，从源头上预防和治理腐败。坚决治理损害群众利益的突出问题，严肃查处各种违法违纪案件，以优良政风凝聚民心。

　　各位代表，我们已经踏上了“十二五”新的征程。神圣使命激励我们勇往直前，人民期望不容我们丝毫懈怠。让我们更加紧密地团结在以胡锦涛同志为总书记的党中央周围，高举中国特色社会主义伟大旗帜，在中共铁岭市委的正确领导下，乘胜奋进，继往开来，为早日实现新铁岭大铁岭宏伟目标而努力奋斗！
